# Efficacy and safety of pharmacological treatments for Lyme neuroborreliosis in children: a systematic review

**DOI:** 10.1186/s12883-016-0708-y

**Published:** 2016-09-29

**Authors:** Rick Dersch, Tilman Hottenrott, Stefanie Schmidt, Harriet Sommer, Hans-Iko Huppertz, Sebastian Rauer, Joerg J. Meerpohl

**Affiliations:** 1 Cochrane Germany, Medical Center-University of Freiburg, Berliner Allee 29, D-79110 Freiburg im Breisgau, Germany; 2 Department of Neurology, Medical Center-University of Freiburg, Breisacher Str. 64, D-79106 Freiburg, Germany; 3 UroEvidence@Deutsche Gesellschaft für Urologie, Berlin, Germany; 4 Institute of Medical Biometry and Statistics, Medical Center-University of Freiburg, Stefan-Meier-Str- 26, D-79104 Freiburg, Germany; 5 Prof.-Hess-Kinderklinik, Klinikum Bremen-Mitte, Sankt-Jürgen-Str. 1, D-28177 Bremen, Germany; 6 Centre de Recherche Épidémiologie et Statistique INSERM Sorbonne Paris, Cochrane France, Hôpital Hôtel-Dieu, 1 place du Parvis Notre Dame, 75181 Paris Cedex 04, France

## Abstract

**Background:**

Many aspects of pharmacological treatment of Lyme neuroborreliosis in children, such as choice of drug, dosage, and duration are subject to intense debates, leading to uncertainties in patients’ parents and healthcare providers alike. To assess the available evidence for pharmacological treatment for children with Lyme neuroborreliosis we conducted a systematic review.

**Methods:**

The comprehensive systematic literature search included randomized-controlled trials (RCTs) and non-randomized studies (NRS) on treatment of Lyme neuroborreliosis in children (age <18 years). Our primary outcome was neurological symptoms after treatment. Risk of bias was assessed with the Cochrane risk of bias tools for RCTs and NRS. Quality of evidence was assessed using the Grading of Recommendations Assessment, Development and Evaluation (GRADE) approach.

**Results:**

Two RCTs and four NRS were eligible for inclusion. Risk of bias in RCTs and NRS was generally high. Reporting of studies was generally poor. Regarding the primary outcome neurological symptoms at 1–3 months, no statistically significant difference could be found in cohort studies between doxycycline and beta-lactam antibiotics. In two RCTs comparing penicillin G and ceftriaxone, no patient experienced residual neurological symptoms at the last reported time points. Quality of evidence according to GRADE was judged very low.

**Conclusions:**

Data is scarce and with limited quality. Several issues could not be addressed due to scarcity of information. No eligible study compared different treatment durations. According to the available evidence, there seems to be no difference between different antibiotic agents for the treatment of Lyme neuroborreliosis in children regarding neurological symptoms. We found no evidence that supports extended antibiotic regimes.

**Review registration:**

Systematic review registration: CRD42014008839.

**Electronic supplementary material:**

The online version of this article (doi:10.1186/s12883-016-0708-y) contains supplementary material, which is available to authorized users.

## Background

Lyme borreliosis is a tick-borne infectious disease affecting several organ systems including the nervous system caused by Borrelia burgdorferi sensu lato. It occurs with an incidence of about 111/100,000 inhabitants in Central Europe, of which approximately 2–3 % will develop neurological manifestations [1, 2], based on the large population-based study from central Germany. A bimodal pattern with two age peaks can be observed, one in children aged 5–14 years and one in elderly people aged 65–74 years [2, 3]. By far the most frequent early manifestation of Lyme borreliosis is erythema migrans, although other manifestations of the disease can occur without dermal signs [4].

Common clinical manifestations of Lyme neuroborreliosis in children are cranial nerve palsy and meningitis [3, 5]. Late manifestations, like myelitis or encephalitis, are rarely seen in children [6]. Diagnosis is usually based on clinical presentation, serologic testing and analysis of cerebrospinal fluid [4].

The likelihood of a diagnosis of Lyme neuroborreliosis in adult patients can be evaluated by case definitions regarding the available diagnostic results [7, 8]. According to results of diagnostic tests, likelihood of diagnosis can be rated as ‘possible’, ‘probable’ or ‘definite’ as proposed by the European Federation of Neurological Societies [8, 9]. Unfortunately, no validated case definitions exist for children. The qualification as a ‘possible’ case requires only the detection of antibodies against Borrelia burgdorferi in serum, which is reported to occur in approximately 5 % of otherwise healthy children in endemic areas [10]. The case definition of a ‘probable’ Lyme neuroborreliosis requires a confirmed lymphocytic pleocytosis in the CSF analysis with no other plausible explanation. The case definition of a ‘definite’ Lyme neuroborreliosis is determined by the presence of specific intrathecal antibodies against Borrelia burgdorferi additional to a conformed lymphocytic pleocytosis in CSF analysis. In early stages of Lyme neuroborreliosis in children, an intrathecal synthesis of antibodies against Borrelia burgdorferi in CSF may be absent [11]. If untreated, acute symptoms may vanish in some cases, but some children with Lyme neuroborreliosis may develop late manifestations of the musculoskeletal or nervous systems [12, 13].

Residual symptoms and long term outcomes after treatment are subjects to debate, as some studies report cognitive deficits in children with Lyme neuroborreliosis [14], whereas other studies report normal findings in neuropsychological tests for children with Lyme neuroborreliosis after antibiotic treatment [15].

Choice of drug, route of administration, and duration of treatment are still a matter of debate [16, 17]. Treatments with multiple antibiotic drugs concomitantly, antibiotic agents like carbapenems and adjuvant drugs like hydroxychloroquine and extended antibiotic treatments >28 days are recommended by guidelines from patient advocacy groups [17]. Extended antibiotic treatments may cause considerable harm, even fatal complications have been reported [18, 19]. Furthermore, unnecessary extended antibiotic courses contribute to the growing problem of multidrug-resistant bacteria [20].

Guidelines from scientific societies recommend treatment for 14–28 days [7, 16, 21]. Recommendations for such treatments should be based on solid clinical evidence. We assessed the available evidence for pharmacological treatments for children with Lyme neuroborreliosis.

## Methods

We searched the databases MEDLINE (via Ovid, from 1950 to 2015), EMBASE (via Scopus, from 1980 to the present) and the Cochrane Central Register of Controlled Trials for eligible studies. Search strategies are shown in Additional file 1. No language restrictions were set. In addition, we searched three trials registers (www.controlled-trials.com, www.clinicaltrials.gov and www.who.int/trialsearch/) to identify further published or unpublished studies or data for completed or ongoing studies.

We included randomized controlled trials (RCTs) and non-randomized studies (NRSs) evaluating pharmacological treatment of children (age <18 years) with clinically diagnosed Lyme neuroborreliosis. Studies were eligible for inclusion if they reported a comparison of a pharmacological treatment against another pharmacological treatment, against placebo or against no treatment. Studies without a comparison group or a population of less than five patients were excluded.

Diagnosis of Lyme neuroborreliosis was based on the clinical case definition by Halperin, Kaiser and Mygland [7–9]. Diagnostic criteria for case definitions were described in detail in a protocol for a systematic review on pharmacological treatment of Lyme neuroborreliosis in adults [22]. We applied the same methods in this systematic review regarding pharmacological treatment in children. Studies regarding patients with ‘Post-Lyme-Disease’, defined as previously treated patients with persistent symptoms in the absence of evidence for ongoing infection, were excluded. Any pharmacological treatment, including combinations of treatments, was considered. Single agents as well as groups of antibiotics were compared with each other.

Our primary outcome was ‘neurological symptoms after treatment’. If several time points were reported in a primary study, data from the last time point was considered. If data permitted, results were presented for short term follow-up (1 to 4 months following the start of treatment) and for long term follow-up (last reported time point). Secondary outcomes were adverse events, disability, patient reported outcomes (e.g., quality of life, pain, fatigue, depression, cognition and sleep), and cerebrospinal fluid pleocytosis. Any adverse event as defined and reported by the original authors was considered. Adverse events were reported as serious adverse events when they required hospitalization, were life-threatening, fatal or when reported as serious adverse events by the original authors.

Firstly, one reviewer (RD) evaluated titles and abstracts to determine whether the study was possibly eligible. Secondly, each full text was evaluated independently by two reviewers (RD, SS or TH) for eligibility. Disagreements were resolved by discussion or with a third reviewer (JM). Two review authors independently extracted data from the full texts of included studies using a specifically developed extraction form, which had been piloted previously. Data was entered into Review Manager (RevMan 5.3) by one of the reviewers and checked by a second reviewer. Discrepancies in data extraction or entry were resolved by discussion with a third reviewer. Reviewers were not blinded to study author, journal, or institution. Risk of bias was assessed by two reviewers independently using the Cochrane risk of bias (RoB) tools for RCTs and for NRSs, respectively [23, 24]. According to the recommendations for the Cochrane RoB-tool for NRSs, no studies assessed as having a ‘critical’ risk of bias were included in the quantitative data synthesis. We initially planned to assess the primary outcome ‘neurological symptoms’ as a continuous outcome. However, the majority of included studies reported the outcome dichotomized, so the results are presented accordingly. If neurological symptoms were reported as continuous or categorical variables, data was appropriately dichotomized according to the measurement scales and categories used. Data was analyzed on an intention-to-treat basis. If this was not possible, data was used as reported by the primary authors. If data was only available in graphical format, we thoroughly estimated the numeric values. Heterogeneity among studies was investigated by using the Chi^2^ test and I^2^ test, see protocol for details [22]. Risk for publication bias was reduced in our systematic review by ensuring a comprehensive search for eligible studies including three trial registries. Only a small set of studies was available for comparisons, so we did not provide a funnel plot.

Pooling of data and meta-analysis of studies was only considered among studies with similar design (e.g., RCTs were only combined with other RCTs) and limited heterogeneity. Combined estimates were not provided for studies with considerable differences in the included population or differences regarding interventions. We planned to provide estimates of treatment effects on a fixed effect model. We planned to calculate pooled risk ratios and 95 % confidence interval across comparable studies using Review Manager (RevMan 5.3). Subgroup analyses were planned to consider dosage of drugs, geographical origin of studies, length of treatment, and likelihood of diagnosis. Sensitivity analyses were planned to assess the effect of risk of bias in included studies. We used the Grading of Recommendations Assessment, Development and Evaluation (GRADE) approach to assess the quality of evidence for each outcome [25].

## Results

**Fig. 1:**
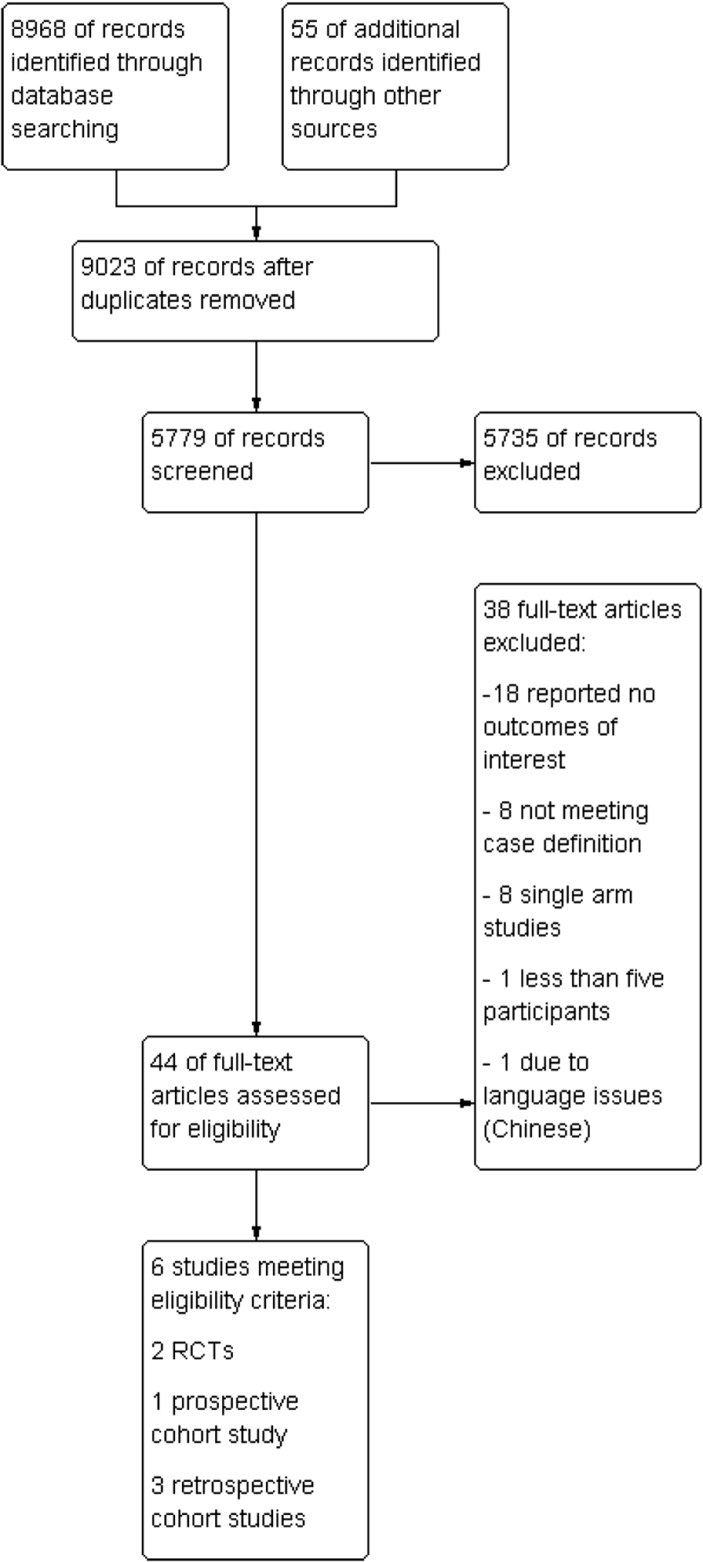
PRISMA flow chart

The search identified 5779 bibliographic records after removal of duplicates, of which 5735 were excluded, and 44 full-text articles were retrieved for detailed examination (Fig. 1). We identified six eligible studies, two RCTs [26, 27], and four NRS (one prospective cohort study [28] and three retrospective cohort studies [29–31]. Reasons for exclusion of the remaining 38 studies are listed in Fig. 1. One retrospective cohort study reported on patients with ‘probable’ Lyme neuroborreliosis [30], all other studies included patients according to the ‘possible’ case definition.

The included studies compared different interventions, which are summarized in Table 1. We merged intervention groups with betalactam antibiotics together to be compared to patients treated with doxycycline. Three studies compared different kinds of betalactam antibiotics [26–28], one evaluated betalactam antibiotics and doxycycline [30], whereas two studies investigated a variety of interventions [29, 31].

**Table 1:** Characteristics of included studies

Study	Sample size	Case definition	Interventions	Length of treatment	Setting	Country
RCT						
Millner 1995	41	possible	penicillin G 300,000-375,000 IU/kg, ceftriaxone 100 mg/kg, sample size of groups not reported	14 days	Tertiary care center	Austria
Müllegger 1991	23	possible	penicillin G 400,000-500,000 IU/kg (*n* = 11), ceftriaxone 75–93 mg/kg (*n* = 12)	14 days	Tertiary care center	Austria
Prospective cohort studies						
Jörbeck 1987	9	possible	penicillin G 150 mg/kg (*n* = 8), cefuroxime 4.5 g (*n* = 1)	10–19 days	Tertiary care center	Sweden
Retrospective cohort studies						
Thorstrand 2002	203	probable	ceftriaxone 100 mg/kg, maximum 2 g (*n* = 109), penicillin 100 mg/kg (*n* = 53), doxycycline 4 mg/kg, maximum 200 mg (*n* = 22), cefotaxime 100 mg/kg (*n* = 19)	10 days	Tertiary care center	Sweden
Bingham 1995	19	possible	ceftriaxone, amoxicillin, erythromycin, penicillin, doxycycline, steroids, aciclovir or no treatment. Dosages not stated.	14–30 days	Tertiary care center	USA
Skowronek-Bala 2008	9	possible	ceftazidim + doxycycline (*n* = 5), amoxicillin + doxycycline (*n* = 1), ceftazidim + amoxicillin (*n* = 1), doxycycline (*n* = 1), ceftazidim (*n* = 1)	3–6 weeks	Tertiary care center	Poland

The most frequent interventions were penicillin G (five studies), ceftriaxone (four studies) and doxycycline (two studies). No eligible studies evaluating treatment with hydroxychloroquine, azithromycin, minocycline or carbapenem antibiotics were identified. Length of treatment was 14 days in both RCTs. NRS showed considerable differences regarding length of treatment ranging from 10 to 30 days.

**Fig. 2:**
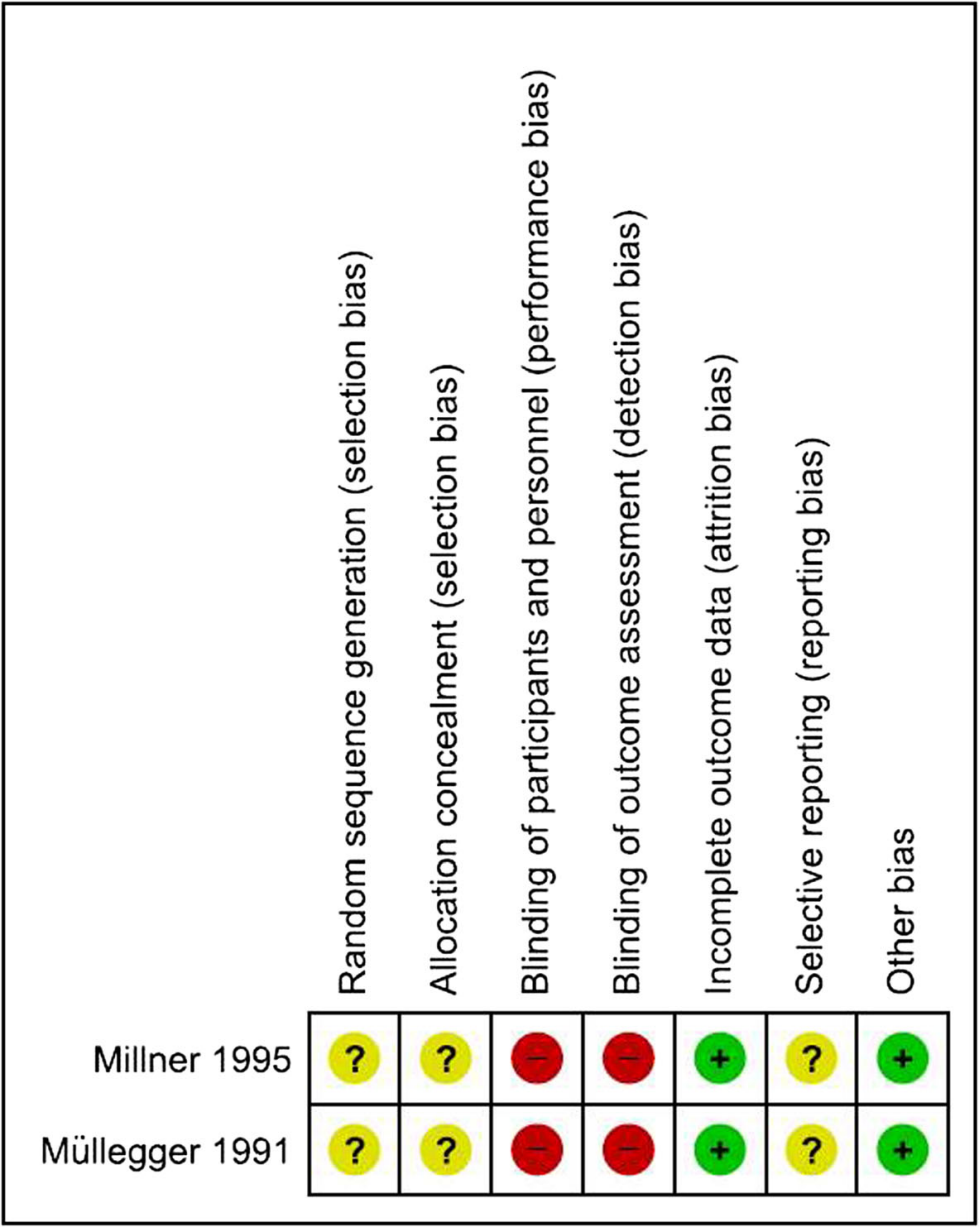
Risk of bias in randomized controlled trials

**Fig. 3:**
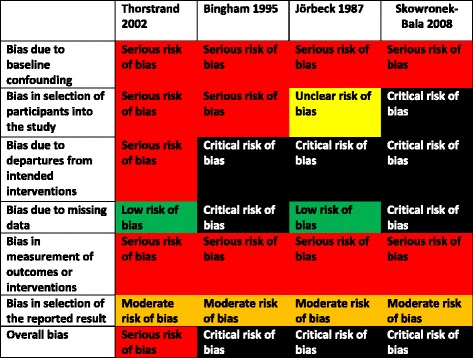
Risk of bias in non-randomized studies

All studies had serious risk of bias issues (Fig. 2). RCTs suffered from poor reporting on allocation concealment, random sequence generation and lack of blinding. Risk of bias and poor reporting were even more problematic in NRS (Fig. 3). Sample bias, baseline confounding and blinding of outcome assessment were major issues in all studies. Interventions were described insufficiently in three NRS [28, 29, 31]. All expect one NRS had ‘critical’ overall risk of bias [30]. Selective reporting cannot be ruled out in any RCT or NRS, as no corresponding protocols were available.

**Fig. 4:**
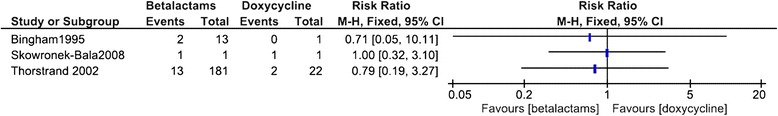
Forest-plot for the comparison of betalactam antibiotics versus doxycycline for residual neurological symptoms in non-randomized studies

Data on neurological symptoms after pharmacologic treatment for the comparison of betalactam antibiotics against doxycycline could be extracted from three NRS [30, 31]. As two of these studies had critical risk of bias, data were not pooled [31]. Estimates from all studies had wide confidence intervals and showed no statistically significant difference between the two treatments (Fig. 4). No data regarding our secondary outcomes could be extracted.

**Fig. 5:**
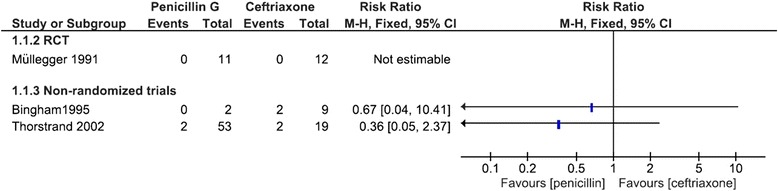
Forest-plot for the comparison of penicillin G versus ceftriaxone for residual neurological symptoms

Data on neurological symptoms after pharmacologic treatment for the comparison of penicillin G against ceftriaxone could be extracted from one RCT and two NRS [26, 28, 30]. No estimate could be provided for the RCT, as no patients had neurological symptoms at last reported time point after treatment. The NRS could not be pooled due to critical risk of bias. Estimates from single studies had wide confidence intervals due to small sample sizes and showed no statistically significant differences between treatments (Fig. 5). Millner and colleagues compared treatment with penicillin G and ceftriaxone in an RCT, but did not provide data on sample sizes in treatment groups, so study data could not be combined with that of Müllegger and colleagues [26, 27]. The authors state that at last reported follow up (12 months after treatment) no participant had neurological symptoms in either treatment group. Millner and colleagues reported no adverse events for the penicillin G group, but moderate allergic skin rash (*n* = 1), elevated liver enzymes (*n* = 2), and asymptomatic bile concrements (*n* = 6) in the ceftriaxone group. Bile concrements were actively sought via sonography in the ceftriaxone group but not in the penicillin G group, which diminishes comparability. Adverse events reported in other studies could not be attributed to an intervention, so no comparison could be performed. No data regarding other secondary outcomes could be extracted.

**Fig. 6:**
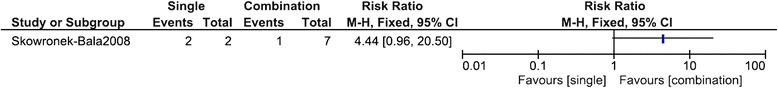
Forest-plot for the comparison of combinations of antibiotic treatments versus single drugs on residual neurological symptoms

One retrospective cohort study with critical risk of bias reported data on neurological symptoms after treatment with combinations of antibiotic treatments against single drugs. The difference between these two heterogeneous groups was not statistically significant (RR 4.44, 95 % CI 0.96-20.50, *p* = 0.0558, Fig. 6).

None of our pre-planned subgroup and sensitivity analyses considering dose, geographical origin, length of treatment, likelihood of diagnosis, or risk of bias were possible for any comparison due to the paucity of data.

The quality of evidence according to GRADE is presented in Tables 2, 3 and 4 [25]. Quality of evidence in RCTs as well as in NRS was very low, considerably lowering the overall confidence in the presented results. The reasons for down-rating the quality besides risk of bias in RCTs were risk of bias and imprecision, in NRS risk of bias, indirectness, and imprecision.

**Table 2:** GRADE evidence table for the comparison betalactam antibiotics vs. doxycycline for children with Lyme neuroborreliosis

Quality assessment						№ of patients		Effect	Quality
№ of studies	Study design	Risk of bias	Inconsistency	Indirectness	Imprecision	Betalactam antibiotics	Doxycycline	Relative (95 % CI)	
Neurological symptoms at last reported time point									
3	observational studies	very serious^a^	not serious	serious^b^	serious^c^	15/195 (7.7 %)	3/25 (12.0 %)	not pooled	⨁◯◯◯ VERY LOW

^a^baseline confounding, selected patients, lack of blinding, interventions insufficiently described

^b^heterogeneous interventions, interventions not clearly described

^c^small sample size, optimal information size not met

**Table 3:** GRADE evidence table for the comparison penicillin vs. ceftriaxone for children with Lyme neuroborreliosis

Quality assessment						№ of patients		Effect	Quality
№ of studies	Study design	Risk of bias	Inconsistency	Indirectness	Imprecision	Penicillin	Ceftriaxone	Relative (95 % CI)	
Neurological symptoms at last reported time point									
1	randomised trials	serious^a^	not serious	not serious	very serious^b^	0/11 (0.0 %)	0/12 (0.0 %)	not estimable	⨁◯◯◯ VERY LOW
Neurological symptoms at last reported time point									
2	observational studies	very serious^c^	not serious	serious^d^	serious^e^	2/55 (3.6 %)	4/28 (14.3 %)	not pooled	⨁◯◯◯ VERY LOW

^a^no blinding, randomisation and allocation concealment not stated appropriately, selective outcomes reporting cannot be excluded

^b^very small sample size, optimal information size not met

^c^baseline confounding, selected patients, lack of blinding, interventions insufficiently described

^d^heterogeneous interventions, interventions not clearly described

^e^small sample size, optimal information size not met

**Table 4:** GRADE evidence table for the comparison combination of antibiotics vs. single drugs for children with Lyme neuroborreliosis

Quality assessment						№ of patients		Effect	Quality
№ of studies	Study design	Risk of bias	Inconsistency	Indirectness	Imprecision	Combination of antibiotics	Single drugs	Relative (95 % CI)	
Neurological symptoms at last reported time point									
1	observational studies	very serious^a^	not serious	serious^b^	serious^c^	1/7 (14.3 %)	2/2 (100.0 %)	RR 4.44 (0.96 to 20.50)	⨁◯◯◯ VERY LOW

^a^critical risk of bias according to ARCOBAT NRSI due to baseline confounding, selected patients, lack of blinding, interventions insufficiently described

^b^heterogeneous interventions applied

^c^small sample size, optimal information size not met

## Discussion

A strength of this review is the comprehensive search of the available literature, including three databases for clinical trials and screening the reference list of included studies, minimizing the risk of bias in study selection. Due to the small number of studies included in these meta-analyses and considerable risk of bias, only limited conclusions can be drawn from them. We were unable to assess publication biases due to the low number of available studies for each of the comparisons.

Literature on pharmacological treatment of Lyme neuroborreliosis in children is scarce and with very limited quality. Most of the available studies were performed several decades ago when pharmacologic treatment was less rigorously assessed as it would be performed today in case of new drugs or indications.

The available evidence is insufficient to identify relevant differences between the evaluated antibiotics. However, due to small sample sizes and resulting imprecision in eligible studies, clinically relevant differences between treatments cannot be excluded.

Different strains of Borrelia burgdorferi show different patterns of geographical distribution [32]. Although five of six included studies were performed in Europe, results from this review may be also applicable in regions where distribution of these strains is different, as in North America, since neurological manifestations differ in frequency but not in clinical presentation. Only one study used the ‘probable’ case definition, whereas the other studies applied diagnostic criteria consistent with the ‘possible’ case definition. As this category often lacks CSF analysis, it is more broad compared to the other case definitions and therefore less specific. More ‘false positive’ patients may be included in studies using the “possible” case definition, probably diminishing treatment effects.

Currently an ongoing Cochrane review evaluates pharmacologic treatments for Lyme neuroborreliosis [33]. As treatment of Lyme neuroborreliosis in children is not the focus of the ongoing Cochrane review, applicability of its results on a pediatric population may be low.

Prognosis of Lyme neuroborreliosis in children after treatment appears to be good, irrespective of the antibiotic regimen studied in these trials. Unfavorable outcomes or poor treatment responses were infrequent regardless of which antibiotic was used. This is illustrated by the wide confidence intervals in pooled estimates due to paucity of respective unfavorable events.

Interpreting a limited body of evidence and drawing implications for clinical practice is difficult. No statistically significant difference was found between beta-lactam antibiotics and doxycycline regarding neurological symptoms after treatment. However, a relevantly lower rate of neurological symptoms for either drug cannot be excluded due to wide confidence intervals. Unfortunately, no data on adverse events were reported for this comparison, so we are unable to provide evidence-based conclusions regarding drug safety.

Doxycycline and beta-lactam antibiotics are recommended as alternative treatments by many guidelines, although use of doxycycline is usually not recommended for children <9 years because of the potential for impairing dental development [7, 16].

No statistically significant difference was found between penicillin G and ceftriaxone regarding neurological symptoms after treatment. Again, a relevantly lower rate of neurological symptoms for either drug cannot be excluded due to wide confidence intervals. Adverse events were only reported for children treated with ceftriaxone in one study (*n* = 9), but confidence in this result is rather low. Severity of these reported adverse events was generally mild and therefore may only partially influence treatment choice. Concordant with these findings, both penicillin G and ceftriaxone are recommended for treatment of Lyme neuroborreliosis in children by several guidelines [7, 16, 21].

No statistically significant difference was found between combination of antibiotics and single drugs. Confidence in this result is very low, as data was only reported in one retrospective cohort study with a small sample size (*n* = 9) with heterogeneous treatments, follow-up periods and critical risk of bias issues. Therefore, no convincing evidence for combination of antibiotics being superior to treatments with single antibiotics could be identified. This is in contrast with recommendations for combination treatments from guidelines developed by patient advocacy groups [17], but is in agreement with recommendations of guidelines from scientific societies [16]. This finding is also in concordance with the evidence from available studies on treatment of Lyme neuroborreliosis in adults [34].

However, besides the very low quality of the available evidence and the lack of recent studies, it remains noteworthy for which interventions no evidence from eligible studies could be found at all. No studies comparing extended antibiotic treatments to treatments of 10–21 days could be found. No eligible studies evaluating treatment with macrolide antibiotics, antimalarial drugs or carbapenems in children with Lyme neuroborreliosis could be identified. The guideline recommendations for these treatment regimens from patient advocacy groups are not based on any published evidence [17].

As the provided estimates suffer from considerable imprecision, it is likely that results of future clinical trials on this topic will change the presented results. Based on the available evidence, most questions arising in clinical practice remain unanswered.

One issue of considerable interest is whether oral treatment with doxycycline may be sufficient to treat early forms of Lyme neuroborreliosis including facial palsy with lymphocytic pleocytosis in children >8 years. No studies evaluated extended antibiotic treatment regimes. As long as such a treatment is not evaluated in a high quality trial, recommendations favoring such regimes remain ill-founded. The scarcity of high quality trials and the lack of recent studies is rather surprising. There is clearly a need for large high quality trials evaluating pharmacological treatments for Lyme neuroborreliosis in children. The included population should be adequately described according to consensus derived case definitions [35]. Trials should be registered prior to enrolment of the first patient and use predefined outcomes. However due to the good clinical results of present treatments used during the last 30 years in children it might be difficult to find a sponsor for large high quality trials evaluating pharmacological treatments for Lyme neuroborreliosis in children.

## Conclusion

According to the available evidence, there seems to be no difference between different antibiotic agents for the treatment of Lyme neuroborreliosis in children regarding neurological symptoms. No eligible study compared different treatment durations. We found no evidence that supports extended antibiotic regimes.

## Additional file

Additional file 1:
**Appendix 1.** Ovid MEDLINE Search Strategy. **Appendix 2.** SCOPUS Search Strategy. **Appendix 3.** CENTRAL Search Strategy. (DOCX 15.4 kb)

## Abbreviations

CI: Confidence interval
CSF: Cerebrospinal fluid
GRADE: Grading of Recommendations Assessment, Development and Evaluation
NRS: Non-randomized studies
RCT: Randomized controlled trial
RoB: Risk of bias
RR: Relative risk

## References

[1] Huppertz HI, Böhme M, Standaert SM, Karch H, Plotkin SA (1999). Incidence of Lyme borreliosis in the Würzburg region of Germany. Eur J Clin Microbiol Infect Dis Off Publ Eur Soc Clin Microbiol.

[2] Robert Koch Institut. Meldepflicht für Lyme-Borreliose in Bayern – eine erste Bilanz. Epidemiol Bull. 2015;23:56-62. (Nr. 8).

[3] Lohr B, Müller I, Mai M, Norris DE, Schöffski O, Hunfeld K-P (2015). Epidemiology and cost of hospital care for Lyme borreliosis in Germany: lessons from a health care utilization database analysis. Ticks Tick-Borne Dis.

[4] Huppertz HI (1990). Childhood Lyme borreliosis in Europe. Eur J Pediatr.

[5] Skogman BH, Croner S, Nordwall M, Eknefelt M, Ernerudh J, Forsberg P (2008). Lyme neuroborreliosis in children: a prospective study of clinical features, prognosis, and outcome. Pediatr Infect Dis J.

[6] Wilke M, Eiffert H, Christen HJ, Hanefeld F (2000). Primarily chronic and cerebrovascular course of Lyme neuroborreliosis: case reports and literature review. Arch Dis Child.

[7] Halperin JJ, Shapiro ED, Logigian E, Belman AL, Dotevall L, Wormser GP (2007). Practice parameter: treatment of nervous system Lyme disease (an evidence-based review): report of the Quality Standards Subcommittee of the American Academy of Neurology. Neurology.

[8] Kaiser R (1998). Neuroborreliosis. J Neurol Mai.

[9] Mygland A, Ljøstad U, Fingerle V, Rupprecht T, Schmutzhard E, Steiner I (2010). EFNS guidelines on the diagnosis and management of European Lyme neuroborreliosis. Eur J Neurol.

[10] Dehnert M, Fingerle V, Klier C, Talaska T, Schlaud M, Krause G (2012). Seropositivity of Lyme borreliosis and associated risk factors: a population-based study in Children and Adolescents in Germany (KiGGS). PLoS One.

[11] Huppertz HI, Horneff G, Neudorf U, Karch H (1994). Acute childhood neuroborreliosis with a selective immune response to a low molecular weight protein expressed by Borrelia garinii. Eur J Pediatr.

[12] Szer IS, Taylor E, Steere AC (1991). The long-term course of Lyme arthritis in children. N Engl J Med.

[13] Bensch J, Olcén P, Hagberg L (1987). Destructive chronic borrelia meningoencephalitis in a child untreated for 15 years. Scand J Infect Dis.

[14] Tager FA, Fallon BA, Keilp J, Rissenberg M, Jones CR, Liebowitz MR (2001). A controlled study of cognitive deficits in children with chronic Lyme disease. J Neuropsychiatry Clin Neurosci.

[15] Zotter S, Koch J, Schlachter K, Katzensteiner S, Dorninger L, Brunner J (2013). Neuropsychological profile of children after an episode of neuroborreliosis. Neuropediatrics.

[16] Wormser GP, Dattwyler RJ, Shapiro ED, Halperin JJ, Steere AC, Klempner MS (2006). The clinical assessment, treatment, and prevention of lyme disease, human granulocytic anaplasmosis, and babesiosis: clinical practice guidelines by the Infectious Diseases Society of America. Clin Infect Dis Off Publ Infect Dis Soc Am.

[17] Cameron D, Gaito A, Harris N, Bach G, Bellovin S, Bock K (2004). Evidence-based guidelines for the management of Lyme disease. Expert Rev Anti Infect Ther.

[18] Holzbauer SM, Kemperman MM, Lynfield R (2010). Death due to community-associated Clostridium difficile in a woman receiving prolonged antibiotic therapy for suspected lyme disease. Clin Infect Dis Off Publ Infect Dis Soc Am.

[19] Patel R, Grogg KL, Edwards WD, Wright AJ, Schwenk NM (2000). Death from inappropriate therapy for Lyme disease. Clin Infect Dis Off Publ Infect Dis Soc Am.

[20] Gootz TD (2010). The global problem of antibiotic resistance. Crit Rev Immunol.

[21] Hobusch D, Christen HJ, Huppertz HI, Noack R (1999). Diagnosis and therapy of Lyme borreliosis in children. Practice guideline of the German Society for Pediatric Infectious Diseases. Klin Padiatr.

[22] Dersch R, Freitag MH, Schmidt S, Sommer H, Rücker G, Rauer S (2014). Efficacy and safety of pharmacological treatments for neuroborreliosis--protocol for a systematic review. Syst Rev.

[23] Higgins J, Green S. Cochrane Handbook for Systematic Reviews of Interventions. Version 5.1.0 [updated March 2011]. The Cochrane Collaboration, 2011. Available from http://handbook.cochrane.org/.

[24] Sterne J, Higgins J, Reeves B. A Cochrane Risk Of Bias Assessment Tool: for Non-Randomized Studies of Interventions (ACROBATNRSI), Version 1.0.0 2014 [cited 2014 19.Dec]. Available from: http://www.riskofbias.info

[25] Guyatt G, Oxman AD, Akl EA, Kunz R, Vist G, Brozek J (2011). GRADE guidelines: 1. Introduction-GRADE evidence profiles and summary of findings tables. J Clin Epidemiol.

[26] Müllegger RR, Millner MM, Stanek G, Spork KD (1991). Penicillin G sodium and ceftriaxone in the treatment of neuroborreliosis in children--a prospective study. Infection.

[27] Millner M (1995). Neurologic manifestations of Lyme borreliosis in children. Wien Med Wochenschr 1946.

[28] Jörbeck HJ, Gustafsson PM, Lind HC, Stiernstedt GT (1987). Tick-borne Borrelia-meningitis in children. An outbreak in the Kalmar area during the summer of 1984. Acta Paediatr Scand.

[29] Skowronek-Bała B, Wesołowska E, Gergont A, Kaciński M (2008). Neuroboreliosis with motoric disturbations in the developmental age. Przegla̧d Lek.

[30] Thorstrand C, Belfrage E, Bennet R, Malmborg P, Eriksson M (2002). Successful treatment of neuroborreliosis with ten day regimens. Pediatr Infect Dis J.

[31] Bingham PM, Galetta SL, Athreya B, Sladky J (1995). Neurologic manifestations in children with Lyme disease. Pediatrics.

[32] Nadelman RB, Wormser GP (1998). Lyme borreliosis. Lancet Lond Engl.

[33] Cadavid D, Auwaerter P, Aucott J, Rumbaugh J. Treatment for the neurological complications of Lyme disease (Protocol). Cochrane Database Syst Rev. 2008. 10.1002/14651858.CD006978.pub2 PMC6463975 27931077

[34] Dersch R, Freitag MH, Schmidt S, Sommer H, Rauer S, Meerpohl JJ (2015). Efficacy and safety of pharmacological treatments for acute Lyme neuroborreliosis - a systematic review. Eur J Neurol.

[35] Stanek G, Fingerle V, Hunfeld K-P, Jaulhac B, Kaiser R, Krause A (2011). Lyme borreliosis: clinical case definitions for diagnosis and management in Europe. Clin Microbiol Infect Off Publ Eur Soc Clin Microbiol Infect Dis.

