# Supplementary material for: Efficacy and safety of pharmacological treatments for Lyme neuroborreliosis in children: a systematic review
Source: BMC Neurol. 2016 Sep 29;16:189. doi: 10.1186/s12883-016-0708-y (PMC5043629; doi:10.1186/s12883-016-0708-y)
Supplement: Additional file 1: — Appendix 1. Ovid MEDLINE Search Strategy. Appendix 2. SCOPUS Search Strategy. Appendix 3. CENTRAL Search Strategy. (DOCX 15.4 kb) [file 12883_2016_708_MOESM1_ESM.docx]

**Appendix 1. Ovid MEDLINE Search Strategy**

1. exp Lyme Disease/

2. lyme*.mp.

3. neuroborreliosis.mp.

4. borreli*.mp.

5. exp Borrelia/

6. (erythem* adj2 migran*).mp.

7. or/1-6

8. exp Brain/

9. brain*.mp.

10. mening*.mp.

11. spinal*.mp.

12. exp Nervous System Diseases/

13. encephal*.mp.

14. radiculi*.mp.

15. radiculo*.mp.

16. Facial Paralysis/

17. facial pal*.mp.

18. facial par*.mp.

19. Myelitis/

20. myel*.mp.

21. (nervous system adj5 dis*).mp.

22. neur*.mp.

23. polyneur*.mp.

24. polyradicul*.mp.

25. mononeur*.mp.

26. (nerve adj5 damage*).mp.

27. (nerve adj5 involvement).mp.

28. bannwarth*.mp.

29. vasculitis/

30. exp vasculitis, central nervous system/

31. vasculiti*.mp.

32. cranial nerve*.mp.

33. or/8-32

34. 7 and 33

**Appendix 2. SCOPUS Search Strategy**

1. TITLE-ABS-KEY(lyme*) OR TITLE-ABS-KEY(neuroborreliosis) OR TITLE-ABS-KEY(borreli*) OR TITLE-ABS-KEY(erythema migrans)

2. TITLE-ABS-KEY(brain*) OR TITLE-ABS-KEY(mening*) OR TITLE-ABS-KEY(spinal*) OR TITLE-ABS-KEY(encephal*) OR TITLE-ABS-KEY(radiculi*) OR TITLE-ABS-KEY(radiculo*) OR TITLE-ABS-KEY(facial pal*) OR TITLE-ABS-KEY(facial par*) OR TITLE-ABS-KEY(myel*) OR TITLE-ABS-KEY(nervous system dis*) OR TITLE-ABS-KEY(neur*) OR TITLE-ABS-KEY(polyneur*) OR TITLE-ABS-KEY(polyradicul*) OR TITLE-ABS-KEY(mononeur*) OR TITLE-ABS-KEY(nerve AND damage*) OR TITLE-ABS-KEY(nerve AND involve*) OR TITLE-ABS-KEY(bannwarth*) OR TITLE-ABS-KEY(vasculiti*) OR TITLE-ABS-KEY(cranial nerve*)

3. 1 AND 2

**Appendix 3. CENTRAL Search Strategy**

1. MeSH descriptor: [Borrelia] explode all trees

2. MeSH descriptor: [Lyme Disease] explode all trees

3. *borreli*

4. erythem* near/2 migran*

5. lyme*

6. 1 OR 2 OR 3 OR 4 OR 5
